# Effects of Effective Microorganism (EM) Inoculation on Co-Composting of *Auricularia heimuer* Residue with Chicken Manure and Subsequent Maize Growth

**DOI:** 10.3390/microorganisms14010106

**Published:** 2026-01-04

**Authors:** Yuting Feng, Yinzhen Zhai, Jiangyan Ao, Keqing Qian, Ying Wang, Miaomiao Ma, Peinan Sun, Yu Li, Bo Zhang, Xiao Li, Han Yu

**Affiliations:** 1 Sanjiang Laboratory, Changchun 130118, China; 15142706595@163.com (Y.F.); 18434865982@163.com (Y.Z.); 19185105621@163.com (J.A.); wy18744105754@163.com (Y.W.); 15865709843@163.com (M.M.); 18911415833@163.com (P.S.); 2 Engineering Research Center of Edible and Medicinal Fungi, Chinese Ministry of Education, Jilin Agricultural University, Changchun 130118, China; 15670567112@163.com (K.Q.); fungi966@126.com (Y.L.); zhangbofungi@126.com (B.Z.)

**Keywords:** effective microorganisms (EM), *Auricularia heimuer* residue, chicken manure, co-composting, lignocellulose degradation, enzyme activity, soil nutrients, maize growth

## Abstract

This study investigated the effects of different Effective Microorganism (EM) inoculation concentrations (0%, 0.5%, 2%, 5%, 10%, 15%) on the co-composting of *Auricularia heimuer* residue with chicken manure and the subsequent growth of maize. The aim was to enhance composting efficiency and promote maize productivity. Results showed that EM addition, particularly at medium concentrations, significantly accelerated the composting process by shortening the heating phase and prolonging the thermophilic period, with the 10% treatment reaching >50 °C by day 2. The 5–10% EM treatments markedly promoted the degradation of cellulose and hemicellulose, and enhanced key enzyme activities (e.g., cellulase and hemicellulase) during composting and maize growth stages. Regarding soil nutrients, the 5% EM treatment led to the most balanced increases in total nitrogen (TN), total phosphorus (TP), and total potassium (TK) contents, with rises of 58.7%, 47.8%, and 130.4%, respectively, during the seedling stage. For maize yield, this treatment enhanced total grain weight, hundred-grain weight, and root activity by 25.7%, 30.9%, and 53.2%, respectively, while also increasing dry matter and root weight. Redundancy and correlation analyses indicated strong positive relationships among root activity, soil TN, cellulase activity, and final yield. In conclusion, EM inoculation at 5–10% optimizes the composting process, improves substrate quality and nutrient supply, and promotes maize root development and yield, with 5% EM offering the most comprehensive benefits. This study provides a practical approach for agricultural waste recycling and sustainable maize cultivation.

## 1. Introduction

Abundant agricultural biomass resources, such as spent mushroom substrate (SMS), animal manure, straw, fruit shells, sawdust, and others, serve as vital renewable materials within ecosystems. According to statistics from the China Edible Fungi Association, China’s annual production of SMS from edible mushroom cultivation has reached approximately 50 million tons [1]. The annual yield of mushroom residue, which is generated after mushroom harvests, is approximately five times that of edible mushrooms [2]. Rich in protein, dietary fiber, minerals, vitamins, and organic compounds such as cellulose, fungal mycelium, and polysaccharides, mushroom residue exhibits physicochemical properties similar to those of peat moss. Its loose, air-permeable structure and strong nutrient-retention capacity make it an excellent organic substrate [3,4,5].

However, the overall utilization rate of SMS remains below 40%. Large quantities are disposed of improperly, not only wasting resources but also causing environmental pollution [3,4]. Promoting the harmless treatment and resource utilization of organic solid waste is, therefore, crucial for reducing environmental impacts and fostering coordinated economic and ecological development.

Aerobic composting is considered an effective waste management approach due to its ability to improve soil structure, promote nutrient transformation, and enhance soil fertility [5]. SMS exhibits low moisture content, low density, and a high carbon-to-nitrogen ratio, whereas livestock manure, such as chicken manure (CM), is characterized by high moisture content, high density, and a low carbon-to-nitrogen ratio [6,7]. Research indicates that co-composting SMS with CM not only optimizes the conversion of organic matter into humus and reduces carbon dioxide emissions but also effectively overcomes the difficulties associated with composting SMS alone [8,9,10]. However, traditional composting still faces challenges, including prolonged fermentation cycles, significant nutrient losses, and incomplete lignocellulose degradation [1,11]. Therefore, developing efficient composting technologies to enhance compost quality and promote sustainable agricultural development holds substantial practical importance.

Microbial inoculants, as live bacterial preparations containing specific functional microorganisms, possess multiple functions such as enhancing compost quality, promoting lignocellulose degradation, increasing enzyme activity, and improving soil properties. Such inoculants have been increasingly adopted as an important biotechnological approach in agricultural production [12,13]. Effective Microorganisms (EM) are composite microbial consortia composed of diverse naturally occurring microorganisms, including photosynthetic bacteria, lactic acid bacteria, yeast, actinomycetes, and fermentative fungi [14]. They are environmentally safe and possess broad ecological functions. Inoculation with EM significantly enhances composting efficiency: beneficial microorganisms rapidly dominate the microbial community, secreting highly active enzymes such as cellulase and protease to accelerate the decomposition of organic matter (especially lignocellulose) [15]. Studies indicate that EM inoculants can also shorten the composting cycle, elevate composting temperatures, promote nitrogen transformation, and enhance humus synthesis [12,16].

Additionally, EM inoculants demonstrate multiple benefits in agricultural applications: improvements in soil structure, enhanced fertility, and promoted biodiversity [17], but also stimulated crop root development, strengthened stress resistance, and increased yield and quality [18,19]. The application of EM inoculants across various crops such as mung beans, wheat, and chili peppers has consistently shown positive effects in boosting production and improving quality [20,21]. Maize (*Zea mays* L.), as a vital grain crop and feed source [22], holds significant importance for national food security due to its high and stable yields. Maize growth is highly sensitive to soil fertility and nutrient supply, with significantly increased demands for nutrients like nitrogen, phosphorus, and potassium during the jointing to tasseling stages [23]. However, long-term reliance on chemical fertilizers has led to soil degradation and a decline in organic matter, severely limiting sustainable maize production [24]. Therefore, exploring the use of high-efficiency organic fertilizers to partially replace chemical fertilizers is crucial for improving soil quality and maize yields.

This study systematically evaluates the comprehensive effects of compost products treated with EM on maize growth, development, and yield formation. However, systematic research on the optimal addition concentration of EM in the specific “SMS-chicken manure” composting system and subsequent effects on crop growth remains limited. In particular, the combined effects of different EM concentrations on the composting process, enzyme activity, nutrient release, and crop growth remain unclear.

Therefore, this study systematically evaluated the effects of EM treatment at different concentrations on the co-composting process of *Auricularia heimuer* residue (a type of spent mushroom substrate, SMS) and chicken manure, lignocellulose degradation, soil nutrient dynamics, and maize growth. The aim was to identify the optimal application concentration of EM and provide a theoretical basis and technical support for the resource utilization of agricultural waste. We propose the following hypotheses:

- EM inoculation significantly accelerates the composting process and enhances compost quality;
- 5–10% EM treatment yields optimal effects on promoting lignocellulose degradation and nutrient release;
- Application of EM-treated compost significantly increases soil enzyme activity, thereby promoting maize growth and yield formation.

## 2. Materials and Methods

### 2.1. Sample Collection and Experimental Design

#### 2.1.1. Composting Fermentation Treatment

The *Auricularia heimuer* residue formula was formulated with 80% wood chips, 15% wheat bran, 2% soybean meal, 1% gypsum, 1% lime, and 1% water. Effective Microorganisms was purchased from Shandong Beijia Biotechnology Co., Ltd. (Weifang, China). The viable bacterial count was ≥10 × 10^8^ cfu·mL^−1^, and the effective microorganisms (EM) primarily included lactic acid bacteria, photosynthetic bacteria, yeasts, etc. For compost preparation, *Auricularia heimuer* residue and chicken manure were mixed at a volume ratio of 8:2 before composting. A total of six compost piles were established, each with a volume of 600 L. This included one control group (without EM addition) and five treatment groups with EM concentrations of 0.5%, 2%, 5%, 10%, and 15%, respectively. Each group received 15 L of EM original liquid, and the initial moisture content was adjusted to 60%. Temperature, moisture content, and compost samples were taken using the five-point sampling method. Five sampling points were established, with one at the center and four around the periphery of the SMS pile; each sampling point was located at half the pile height. A total of 200 g of compost samples are collected daily from each sampling point. Compost temperature was monitored using an alcohol thermometer (Jilin, China) inserted 20 cm into the center of the compost pile. Temperature readings were recorded twice daily at 9:00 AM and 4:00 PM. The collected samples were thoroughly mixed and divided into two portions. One portion was placed in an electric blast drying oven and dried at 40 °C until a constant weight was achieved. The dried samples were subsequently ground and passed through a 40-mesh sieve for analysis of physicochemical properties (pH, total nitrogen, total phosphorus, and total potassium) and lignocellulosic content (cellulose, hemicellulose, and lignin), as well as enzyme activity. The other portion was stored in a –80 °C freezer for enzyme activity analysis. Each treatment was performed with three biological replicates.

#### 2.1.2. Pot-Plant Experiments

The six different compost products prepared in Section 2.1.1 were each mixed with soil at a 4:6 volume ratio for corn container planting. In the control group (CK), 25 kg of cultivation substrate was placed in each pot, and 30 POTS were set up in each treatment group. During the seedling stage and tasseling stage of maize, 500mL of EM solution was applied to each treatment group, respectively. The tested soil was collected from Jilin Agricultural University, and the selected maize variety was Jinboshi 825 (Henan Jinboshi Seed Industry Co., Ltd.) (Zhengzhou, China). All pots were placed in an outdoor experimental site under natural environmental conditions. Water management was uniformly conducted using the weighing method, and weeds were removed promptly to ensure consistent basic growth conditions across all treatments. Rhizosphere soil sampling was collected at the seedling, jointing, tasseling, flowering, and maturity stages of maize using the five-point sampling method. At maturity, three maize plants were randomly selected for whole plant analysis. The collected samples were combined and subsequently divided into two portions. One portion was placed in an electric blast drying oven and dried at a temperature range of 80–90 °C for 20 min. Following this initial drying, the temperature was adjusted to 60 °C and maintained until a constant weight was achieved. The dried samples were subsequently ground, sieved through a 0.5 mm sieve, and stored in a light-protected environment for the analysis of physicochemical properties and lignocellulose content. The remaining portion was stored in a −80 °C freezer for the assessment of enzyme activity.

### 2.2. Determination Methods

#### 2.2.1. Analysis of Compost and Soil Properties

Soil samples were crushed, weighed, and sieved before undergoing digestion with a mixture of sulfuric acid (H_2_SO_4_) and hydrogen peroxide (H_2_O_2_). The digested samples were subsequently diluted to volume, filtered, and analyzed for total phosphorus (TP) using the molybdenum-antimony colorimetric method, total nitrogen (TN) using the Kjeldahl method, and total potassium (TK) using perchloric acid and sulfuric acid digestion methods. Soil pH was determined using the co-potentiometric method, with a water-to-soil ratio of 2.5:1. The cellulose, hemicellulose, and lignin contents were determined by cellulose, hemicellulose, and lignin content assay kits (Suzhou Dream Rhinoceros Biomedical Technology Co., Ltd., Suzhou, China). For enzyme activity analysis, the activities of cellulase, hemicellulase, and lignin peroxidase (Lip) were determined using a specific activity detection kit (Suzhou Dream Rhinoceros Biomedical Technology Co., Ltd., Suzhou, China). The kit numbers are Cellulose (M1733B), Hemicellulose (M1719B), Lignin (M1711B), Cellulase (M1733B), hemicellulase (M1732B), Lignin peroxidase (M1716B). Each treatment was biologically repeated three times.

#### 2.2.2. Analysis of Maize Tissue and Growth Indicators

At corn maturity, tissue samples from different plant parts were collected to measure growth and yield indicators. Pith cellulose content was analyzed by taking pith tissue from the stem, drying and pulverizing it, then using the Cellulose Content Assay Kit (Catalog No. M1733B). Leaf hemicellulose content was determined by drying and pulverizing mature leaves, followed by analysis using the Hemicellulose Content Assay Kit (Cat. No. M1719B). Stem lignin content was measured by drying and pulverizing stem tissue, then analyzing with the Lignin Content Assay Kit (Cat. No. M1711B). Root system vitality was assessed using the TTC reduction method by measuring root tip samples. Yield-related indicators included plant yield (weighed after natural air-drying of grains), hundred-grain weight (average of 100 randomly selected grains), and above-ground dry weight (stems, leaves, and rachis, weighed after drying to constant weight). Root morphological indicators encompass root hair number (manually counted) and fresh weight per root (measured after washing and drying intact roots). Plant morphological indicators include stem diameter (measured at the midpoint of the third internode above the base) and plant height (measured from the stem base to the top of the tassel). All measurements were performed with three biological replicates.

### 2.3. Statistical Analysis

Statistical analyses were conducted using SPSS version 27.0.1, which was employed to calculate the mean and standard error (SE) for lignocellulose content and soil chemical properties across different treatment groups. Redundancy analysis (RDA) was conducted using the Canoco 4.5 software package (Microcomputer Power, Redmond, WA, USA). One-way ANOVA and Duncan’s multiple comparisons were performed using SPSS 27.0.1, with significance levels set at *p* < 0.01 (denoted as **) and *p* < 0.05 (denoted as *). Pearson correlation analyses were conducted in Origin 2025, and correlation heat maps were generated using the same software. Significant correlations were indicated as “*” for *p* < 0.05 and “**” for *p* < 0.01.

## 3. Results

### 3.1. Changes in Temperature, Moisture Content, and pH During Composting

**Figure 1:**
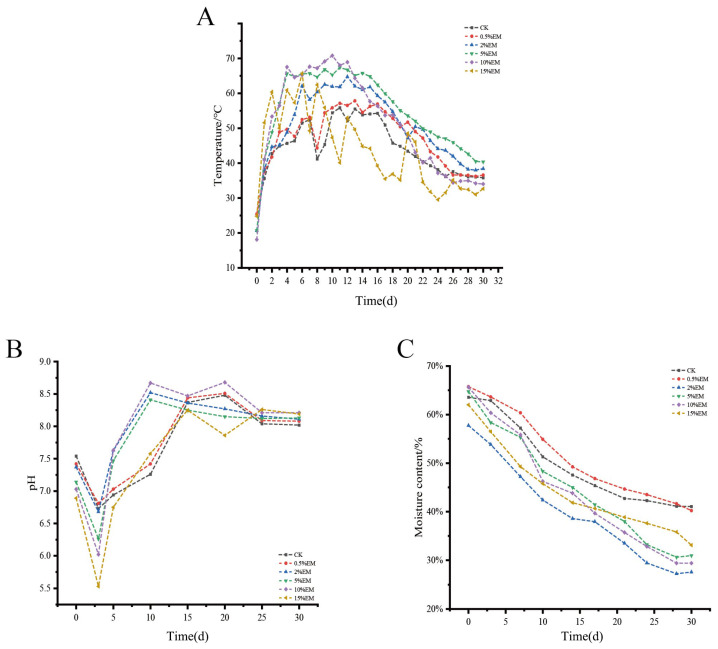
Dynamics of key process parameters during composting. (**A**) Temperature dynamics during composting; (**B**) Compost pH; (**C**) Moisture content.

Figure 1A shows that the entire composting process can be divided into three distinct phases: the initial heating period, the thermophilic period, and the cooling period. Among them, the thermophilic period is defined as the period when the compost temperature reaches or exceeds 50 °C. In the initial phases, the addition of EM shortened the time required for the reactor to reach high temperatures. The control group (CK) had the longest heating duration (7 days), while the inoculated group with a 15% EM concentration had the shortest heating duration (2 days). After entering the thermophilic period, the temperature of each inoculation group rose rapidly to 60 °C. Compared with CK (with a thermophilic period lasting 7 days), the thermophilic period of each EM inoculation group was prolonged by 2 to 9 days, and the average temperature of all inoculation groups was higher than that of CK (54.35 °C). On the 7th, 14th, and 21st days, the pile was turned over. After the temperature of each group remained stable, the fermentation process was basically completed. During the composting period, the 10% EM inoculation group performed the best, rising above 50 °C on the 2nd day. The thermophilic period lasted for 16 days, reaching the highest temperature of 62.28 °C on the 9th day, with an average temperature of 57.22 °C. The temperature decreased steadily in the later stage. The 15% EM group had the shortest heating period and the highest thermophilic period temperature, but this phase was characterized by considerable temperature fluctuations. It may lead to poor stability of the compost. During the composting process, the moisture content of all treatment groups (including CK) in Figure 1C showed a significant decreasing trend throughout the composting period. The moisture content of CK and the treatment groups decreased from 65% at the beginning of composting to about 30% after fermentation. The moisture content of all EM inoculation groups (0.5–15% EM) in the later stage of fermentation was lower than that of CK (41.06%).

As shown in Figure 1B, the pH values of all treatment groups presented a three-stage characteristic of “first decreasing, then increasing, and finally stabilizing” during the composting process. At the beginning of composting, the pH values of each treatment group were close to neutral. However, inoculation with EM significantly altered the duration and intensity of the acidification, recovery, and stabilization phases in each treatment group. From 0 to 3 days, 15% EM acidification was the most intense, with pH plummets from 6.89 to 5.53 (a decrease of 19.7%), a decline far exceeding that of CK (a decrease of 10.7%). Overall, the degree of acidification tended to be more pronounced with higher EM addition, with treatments containing ≥5% EM inducing a significantly stronger (*p* < 0.05) pH decrease than the control during the early stage. During the period of 5 to 15 days, the pH rapidly rose to the alkaline range, with the 2–10% EM group recovering the fastest. On the 10th day, 2% EM (8.67), 5% EM (8.52), and 10% EM (8.41) were significantly higher than CK (7.26). The pH recovery in the 15% EM group lagged relatively. On the 10th day, the pH was 7.58, 0.83 units lower than that in the 10% EM group. At the end of composting, the pH values of each treatment group remained stable at around 8.0, indicating weak alkalinity (*p* < 0.05).

### 3.2. Changes in Lignocellulose Content

**Figure 2:**
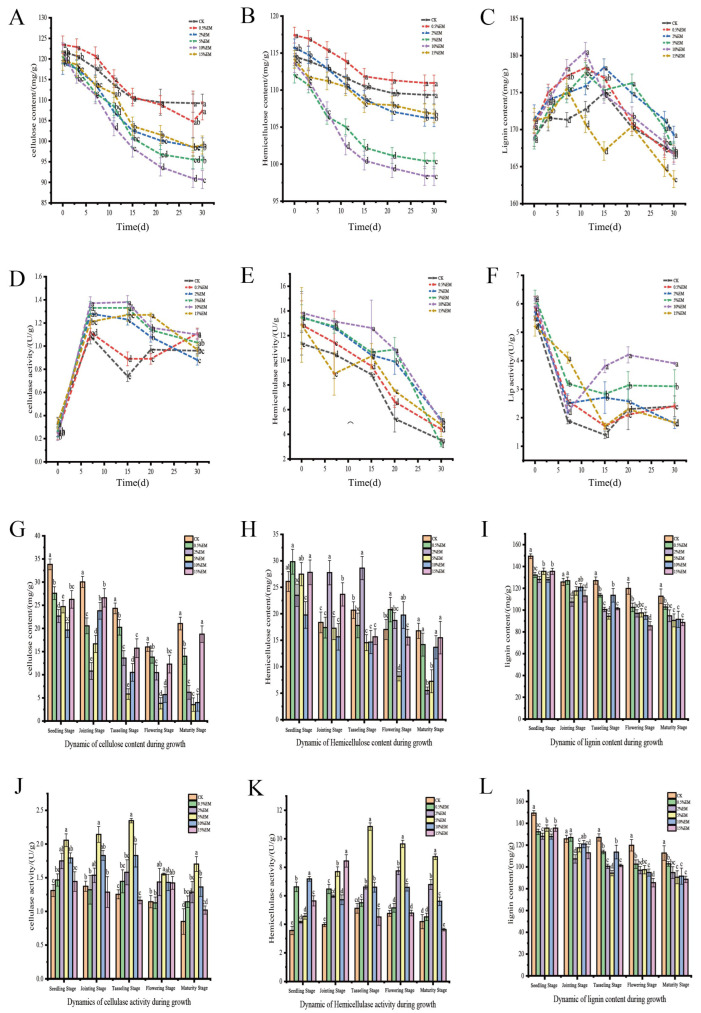
Changes in soil lignocellulose content and lignocellulase activity across different composting treatments. (**A**–**C**) Lignocellulose content during composting; (**D**–**F**) Lignocellulase enzyme activity during composting; (**G**–**I**) Lignocellulose content during the maize growing period; (**J**–**L**) Lignocellulase enzyme activity during the maize growing period. Different lowercase letters indicate significant differences within the same time point at the 0.05 significance level.

Figure 2A–C illustrates that the composting process undergoes two distinct phases of lignocellulose degradation: a rapid degradation phase (approximately 3–15 days) and a slow degradation phase (following approximately 15 days). As shown in Figure 2A, at the initial stage of composting, the cellulose content of the control group (CK) was 121.79 mg/g. The inoculation of EM significantly promoted the degradation of cellulose, and the degradation effect increased with the increase in EM concentration. During the rapid degradation stage, the degradation rates of all inoculation groups reached their highest. Among them, the cellulose content of 5% EM and 10% EM decreased to 111.77 mg/g and 111.33 mg/g, respectively, on the 7th day (5.1% and 5.5% lower than that of CK, respectively). After entering the slow degradation stage, the degradation rate slows down. By the 30th day, the cellulose content in the 15% EM inoculation group dropped to 99.10 mg/g, which was 9.3% lower than CK and, based on statistical analysis at this time point, represented a significantly (*p* < 0.05) greater reduction compared to the other treatments. Considering the degradation dynamics throughout the composting process (including rate and extent), the treatments with 10% to 15% EM exhibited the most pronounced promoting effects on cellulose degradation compared to lower concentrations and the control. Figure 2B shows the degradation trend of hemicellulose, which is quite similar to that of cellulose. In the initial stage, the content of the CK group was 114.57 mg/g, while 0.5% EM (117.42 mg/g) was slightly higher than that of CK. During the high-temperature rapid degradation phase, both the 10% and 5% EM treatments exhibited significantly greater degradation effects comparable to the control group (CK) (*p* < 0.05). During the subsequent stable stage, the degradation rates of all treatment groups tended to level off, but the 5% EM to 15% EM still maintained a relatively high degradation efficiency. Overall, the degradation of hemicellulose was most effectively promoted by the medium-concentration EM treatments. Specifically, both the 5% and 10% EM treatments resulted in significantly lower hemicellulose content compared to the control during the active composting phase, with no statistically significant difference between them. The dynamics of lignin show a trend of “first rising and then falling” (Figure 2B). During the relative enrichment stage, due to the rapid consumption of easily degradable components (such as cellulose and hemicellulose), the relative content of lignin increased: in the CK group, it rose from 168.93 mg/g to 174.95 mg/g, while in the 2% EM microbial agent treatment group, it reached a peak of 178.43 mg/g on the 15th day. After entering the strong degradation stage, refractory substances began to decompose. The CK group decreased to 166.97 mg/g, while the 15% EM inoculation group decreased most significantly to 163.31 mg/g. This result demonstrates that the highest lignin degradation was achieved with the 15% EM treatment. The degradation of lignin is mainly concentrated in the later stage of composting (*p* < 0.05).

Statistical analysis revealed that cellulose content differed significantly among the treatments at multiple key growth stages, including the seedling, jointing, tasseling, and flowering periods. During the entire growth period, the cellulose content decreases in a stepwise manner until it slightly increases at the maturity stage, as shown in Figure 2G. The cellulose content in CK was consistently significantly higher than that in the EM inoculation groups during the seedling, jointing, tasseling, and flowering periods. During the mature period, the cellulose content in the 15% EM treatment group showed a numerical increase compared to CK, but this difference was not statistically significant (*p* > 0.05). The EM inoculation group significantly accelerated the degradation of cellulose. The 5% EM group had the lowest content during the tasseling stage, which was 76.1% lower than that of CK. The concentration of 0.5–2% EM showed significant degradation effects at the seedling stage. 5% EM performed the best from the tasseling stage to the flowering stage, with a degradation rate reaching 76.1%. The 15% EM treatment showed an abnormal increase during the mature period, which was 10.8% lower than CK. The comprehensive degradation rate of 5% EM increased by 45.2% to 76.1%. The degradation effect of hemicellulose content fluctuated significantly during the growth period (Figure 2H). The content is relatively high at the seedling period, fluctuates from the jointing period to the flowering period, and is relatively low at the mature period. During the seedling period, the hemicellulose content in the 15% EM treatment was significantly higher than that in CK, while the 0.5% EM treatment did not differ significantly from CK. During the jointing period and the tasseling period, the 2% EM treatment was significantly higher than CK. During the flowering period, 0.5% EM treatment was significantly higher than CK. During the mature period, CK showed no significant difference from treatments of 0.5% EM, 10% EM, and 15% EM, but was significantly higher than treatments of 2% EM and 5% EM. The 5% EM group reached the lowest value of 8.18 mg/g during the flowering period, which was 52.0% lower than that of CK. The content of 2% EM increases during the jointing period. The 5% EM treatment during the flowering period led to the most significant degradation, while the 15% EM treatment inhibited degradation during the seedling period, and at the mature period, the 10% EM treatment was among the treatments showing the lowest hemicellulose content values.

Lignin shows a continuous degradation trend throughout the entire growth period (Figure 2I), and significant differences in lignin content among treatments were observed at multiple growth stages (though not necessarily between all pairs of treatments within each stage). The 5% EM treatment reached the lowest value during the tasseling period, and the 15% EM treatment exhibited the most significant degradation during the flowering period. Lignin content in the CK treatment remained consistently higher than that in several EM treatments throughout all five growth periods, maintaining relatively elevated levels overall; however, no significant differences were observed between CK and certain treatments. The degradation of each treatment was accelerated during the flowering period. 2% EM had the highest degradation rate at the jointing period, while 5–10% EM maintained efficient degradation during the maturity period. Overall, the 5% EM treatment has the best degradation effect on lignin.

As shown in Figure 2D, cellulase activity during composting exhibited a trend of “rapid increase-peak maintenance-slow decline.” In the initial stage, the enzyme activity of the CK group was 0.34 U/g, which was similar to that of the 15% EM group (0.33 U/g), while the 10% EM treatment had the lowest value (0.22 U/g). Between days 7 and 15, the enzyme activities of all treatment groups increased significantly. The 10% EM treatment reached a peak activity of 1.37 U/g on day 7 (26.1% higher than CK) and remained at 1.38 U/g until day 15. From days 20 to 30, enzyme activity generally decreased. By day 30, the 0.5% EM group (1.11 U/g) and the 10% EM group (1.10 U/g) were still significantly higher than CK (0.96 U/g), while the 2% group had the lowest value (0.88 U/g).

Hemicellulase plays a dominant role in hemicellulose hydrolysis (Figure 2E). Its activity gradually decreased as composting progressed. In the initial stage, the 10% group showed the highest enzyme activity (13.51 U/g), which was 19.1% higher than that of CK (11.34 U/g). On day 7, the 10% EM treatment maintained 13.15 U/g, while the 15% EM treatment decreased sharply to 8.91 U/g (*p* < 0.05), suggesting that high concentrations may inhibit enzyme activity in the early stage. From days 15 to 30, until the end of composting, the 2% EM treatment had the highest enzyme activity (5.2 U/g), followed by the 10% EM treatment (5.01 U/g), and the 5% EM treatment had the lowest activity (3.16 U/g). The CK group remained at a relatively low level throughout. Overall, medium-to-low concentrations of EM treatment maintained a certain level of hemicellulase activity during the mid-to-late stages of composting, whereas high-concentration treatment (15% EM) exhibited a pronounced inhibitory effect.

Changes in Lip activity are shown in Figure 2F. In the early stage of composting, the 5% EM and 10% EM had the highest enzyme activities (6.25 U/g and 6.18 U/g, respectively), which were 12.7–13.6% higher than CK (5.50 U/g). Between days 7 and 20, the 15% EM treatment increased to 4.11 U/g on day 7, while the 10% EM treatment maintained the highest activity in the mid-to-late stages (days 15–30), reaching a peak of 4.21 U/g on day 20. In the later stage, the 10% EM treatment’s enzyme activity (3.91 U/g) was significantly higher than the other groups, followed by the 5% EM treatment (3.10 U/g), while the 2% EM and 15% EM decreased to 1.80–1.83 U/g. These results indicate that the 10% EM treatment consistently maintained the highest lignin-degrading enzyme activity throughout the composting process.

Significant differences in cellulase activity were observed among treatment groups at different growth stages. Cellulase activity fluctuated throughout the growth period (Figure 2J), with higher activity observed during tasseling and flowering stages. Compared to the CK, most EM-treated groups showed significantly enhanced cellulase activity across multiple growth stages, with the stimulatory effect being most pronounced and consistent from the tasseling stage onward. During the booting stage, the 5% EM treatment exhibited a 112.0% increase in enzyme activity compared to CK; at maturity, the 5% EM treatment showed a 109.1% increase relative to CK. Cellulase activity was significantly higher in all EM-treated groups than in CK, with increases ranging from 15.2% to 127.6%. The 10% EM treatment exhibited the highest activity during the seedling stage at 7.187 U/g, representing a 101.5% increase over CK. The 5% EM treatment peaked during the booting stage at 10.857 U/g, showing a 112.0% increase over CK. The 0.5–2% EM treatments significantly enhanced activity during the seedling and jointing stages, while the 5–10% EM treatments showed optimal effects during the tasseling and flowering stages. The 15% EM treatment exhibited inhibition during tasseling and flowering, with activity reduced by 5.4–11.9% compared to CK. The 5–10% EM range was the most suitable, achieving an overall activity increase of 67.3–127.6% across all growth stages.

Hemicellulase activity exhibited a declining trend throughout the growth stages (Figure 2K). Compared to CK, the 5% EM treatment significantly increased hemicellulase activity across all growth stages (increase of 31.0–87.3%), with the most pronounced effect observed during the tasseling stage. The 15% EM treatment exhibited lower activity than CK during the seedling and flowering stages, with enzyme activity increases (4.6–176.3%) significantly lower than cellulase activity. The 5% EM treatment performed optimally across all growth stages, peaking at the tasseling stage with 2.34 U/g, an 87.3% increase over CK. The 5% EM treatment exhibited significantly higher activity than other treatments, while the 0.5–2% EM treatments showed no significant difference from CK (except during the seedling stage). The 15% EM treatment increased activity by 20% compared to CK during the maturity stage, but remained significantly lower than the 5% EM treatment. The 5% EM treatment was the optimal treatment for hemicellulase activity, achieving an average increase of 53.8%.

Lip activity exhibited the most complex response to EM treatment (Figure 2L). High-concentration EM significantly promoted enzyme activity at specific growth stages. Ligninase activity fluctuated throughout the growth period, peaking during the jointing stage and declining during the maturity stage. Compared to CK, 10–15% EM treatments significantly increased enzyme activity during the jointing stage, while the 5% EM treatment significantly increased activity during flowering and ripening. Ligninase activity changes indicated that EM treatment significantly enhanced lignin degradation capacity (increase of 8.8–116.6%), but the optimal concentration varied greatly across growth stages. The 15% EM treatment yielded the highest activity during the seedling and flowering stages (12.28 U/g), while the 0.5% EM treatment was optimal during the tasseling stage (11.16 U/g). The 10% EM treatment exhibited peak activity during the jointing stage (14.77 U/g), but the 15% EM treatment showed a significant decline during the ripening stage, decreasing by 52.0% from its peak.

### 3.3. Changes in Total Nitrogen, Total Phosphorus, and Total Potassium Content

**Figure 3:**
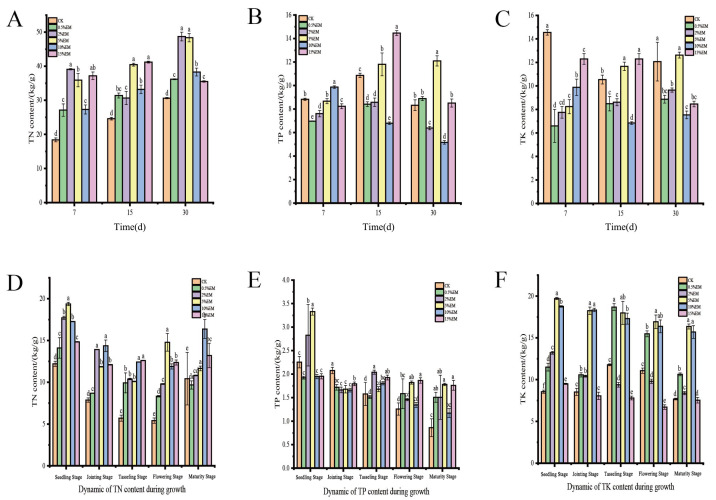
Changes in soil total nitrogen (TN), total phosphorus (TP), and total potassium (TK) under different composting treatments. (**A**–**C**) Changes in TN, TP, and TK contents during composting; (**D**–**F**) Changes in TN, TP, and TK contents during maize growth. Statistical analysis was performed using one-way ANOVA (Duncan’s test, *p* < 0.05). Different lowercase letters are expressed as the same period at the 0.05 level.

In Figure 3A, TN content across different treatments increased significantly with composting duration. The CK group exhibited the lowest TN content, while the 5% EM treatment reached its peak (39.11 g/kg) on day 7, representing a 112.7% increase over the CK group. By day 30, the 2% EM and 5% EM groups reached peak levels (48.71 g/kg and 48.36 g/kg), while the 15% EM group showed a declining trend. Total phosphorus (TP) content changes are shown in Figure 3B. At the mid-composting stage (day 15), the 15% EM group reached its TP peak, significantly higher than the control group, indicating that high-concentration EM promotes phosphorus leaching; During the late composting stage (30 days), TP in the 10% EM group continued accumulating to 12.10 g/kg, while the 15% EM group decreased to 8.51 g/kg. The 2% EM treatment exhibited abnormal TP accumulation (12.10 g/kg) in the late stage. At day 7, the CK treatment showed the highest TK (14.55 g/kg), but all EM treatments exceeded CK as composting progressed. As shown in Figure 3C, at day 15, the TK in the 10% EM treatment significantly increased to 11.66 g/kg, representing a 10.6% increase compared to the CK group. By day 30, the 5% EM and 10% EM treatments maintained higher TK levels (12.62 and 12.07 g/kg).

As shown in Figure 3D, the overall trend of soil TN during the growth period follows a V-shaped curve: higher levels during the seedling stage, decreasing by the flowering stage, and then rising again by the maturity stage. The 5% EM treatment reached peak values at the seedling stage (19.37 g/kg) and flowering stage (14.78 g/kg), exceeding CK by 58.7% and 173.2%, respectively. The 2–5% EM treatments showed the greatest increase from the seedling stage to the jointing stage, with the 2% treatment increasing by 42.1–76.7% compared to CK. The 10–15% EM treatments exhibited a clear advantage from the tasseling stage to the flowering stage, with the 10% EM treatment increasing by 117.8% compared to CK. The 5% EM treatment maintained a relatively high level at maturity, 11.8% higher than CK. Total phosphorus content showed a declining trend (Figure 3E). The 5% EM treatment reached its peak during the seedling stage (3.33 g/kg), 47.8% higher than CK. The 15% EM treatment exhibited the highest content during the flowering stage (1.86 g/kg), 48.3% higher than CK. Treatments ranging from 0.5% to 2% EM performed notably well during the tasseling stage, with the 2% EM treatment increasing by 29.4% compared to CK. 10–15% EM treatments showed significant advantages from flowering to maturity, with the 15% EM treatment increasing by 48.3–104.7% compared to CK; the 5% treatment maintained high levels during the seedling and maturity stages, increasing by 47.8–107.0% compared to CK; the 5% EM treatment increased by 47.8% during the seedling stage and by 107.0% during maturity.

Total potassium content exhibited significant fluctuations (Figure 3F). The 5% EM treatment maintained consistently high levels throughout all growth stages (from 19.71 g/kg at the seedling stage to 16.35 g/kg at maturity), averaging 113.1% higher than CK. The 5% treatment reached its peak content at the seedling stage, exceeding CK by 130.4%; The 10% EM treatment performed excellently from the jointing stage to the tasseling stage, increasing by 115.1–46.9% compared to CK. The 15% EM treatment significantly inhibited potassium release (decreasing by 11.0–39.3% compared to CK at all growth stages), and the 15% EM treatment had significantly lower potassium content than CK at all growth stages. The 5% EM treatment achieved the maximum increase of 130.4% relative to the CK group. The 5% EM treatment was the overall optimal choice, achieving average increases of 81.2%, 107.0%, and 130.4% for TN, TP, and TK, respectively.

### 3.4. Maize Growth Indicators

**Figure 4:**
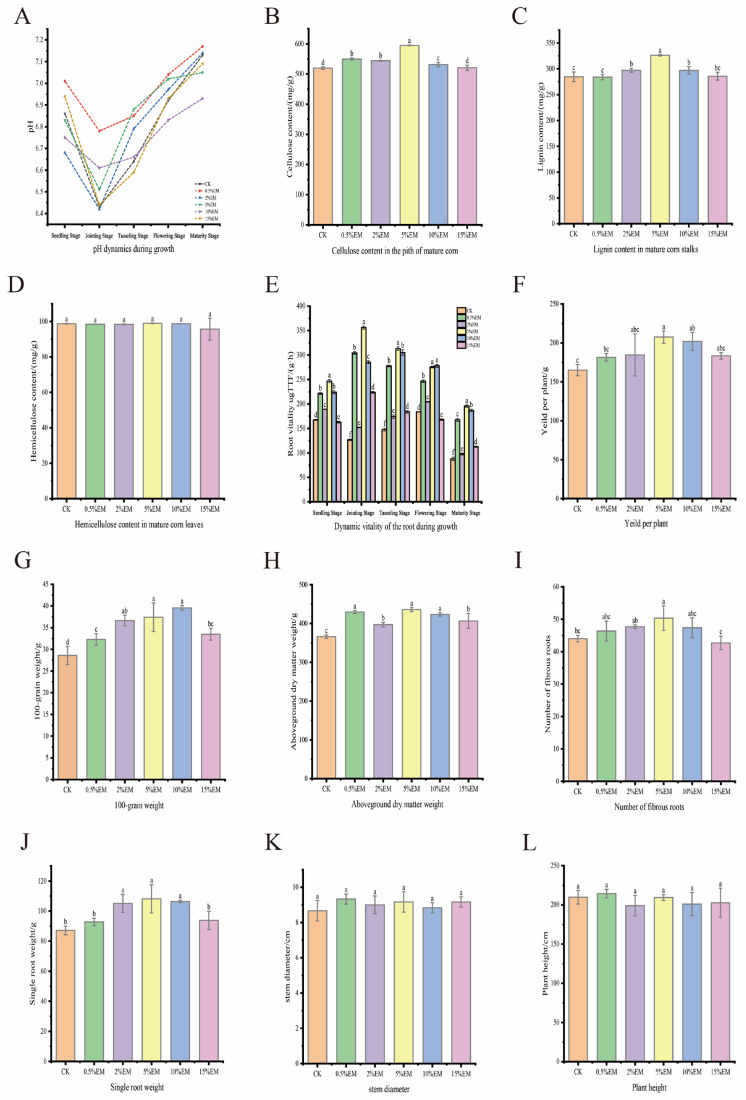
Maize growth indicators: substrate pH during development and agronomic traits at maturity: (**A**) Substrate pH; (**B**) Pith cellulose content; (**C**) Leaf hemicellulose content; (**D**) Stalk lignin content; (**E**) Root activity during maize growth period; (**F**) Yield-per-weight; (**G**) 100-grain weight; (**H**) Aboveground dry matter weight; (**I**) Number of fibrous roots; (**J**) Single root weight; (**K**) Stem diameter; (**L**) Plant height. Different lowercase letters are expressed as the same period at the 0.05 level.

pH values showed a progressive decrease trend across all growth stages (Figure 4A). During the seedling stage, the pH of the CK group was 7.13, while the 5% EM group decreased to 6.95. At maturity, CK was 7.41, and the 5% EM group significantly decreased to 6.90, approaching the neutral range. The pH values of the 10% EM and 15% EM groups were close to or slightly higher than CK during most growth stages. For example, at maturity, the 15% EM group recorded 7.40. The 5% EM group exhibited the lowest pH across all growth stages, particularly at maturity (6.90). In contrast, the pH values of the 10% EM and 15% EM groups were close to or slightly higher than CK during most growth stages.

Pith cellulose content (Figure 4B) indicated that the 5%, 2%, and 0.5% EM treatments formed a group with similarly high values, all of which were higher than the 15% EM treatment. The 5% EM group had the highest content (594.83 mg/g), significantly increasing by 14.4% compared to CK. Treatments with 10% EM and 15% EM showed no significant difference from CK (decreases of 0.1–1.8%); treatments with 0.5% EM and 2% EM increased by 5.7% and 4.7%, respectively. This indicates that 5%EM is the optimal treatment, achieving a 14.4% increase.

Leaf hemicellulose content showed no significant differences among treatment groups compared to CK (Figure 4C), except for an abnormal decrease in the 15% EM group (95.69 mg/g). The 5% EM group was slightly higher than CK, while the 15% EM treatment significantly reduced it by 3.1%.

As shown in Figure 4D, the 5% EM treatment led to a significant increase in stem lignin content compared to the CK. Other EM treatments (2%, 10%, 15%) showed numerically varied but statistically non-significant differences among themselves relative to the 5% EM and CK treatments. The 5% EM group reached the peak value (326.34 mg/g), 14.6% higher than CK; the 2% EM and 10% EM groups increased by 4.4% and 4.3%, respectively; the 0.5% EM and 15% EM treatments showed no difference from CK; the 5% EM treatment promoted the most significant increase in lignin accumulation, with a 14.6% increase.

Root vitality exhibited an inverted U-shaped trend with increasing EM concentration, with 5% being the optimal concentration (peak value of 28.3 U/g). Vitality significantly decreased at concentrations above 5% EM, possibly due to the inhibition of microbial activity by high concentrations of EM (Figure 4E). During the seedling stage, root activity slightly increased to 13.1 ± 0.9 U/g. After adding 0.5% EM, the 5% EM treatment reached its peak (18.6 ± 1.5 U/g), while the CK root activity was 12.3 ± 1.2 U/g, significantly higher than that of other groups. During the jointing stage, CK treatment increased to 15.8 ± 1.1 U/g, while 5% EM and 10% EM treatments significantly rose to 22.4 ± 1.8 U/g and 21.7 ± 1.6 U/g, respectively. Root activity reached its overall maximum during the tasseling stage, with the 5% EM treatment exhibiting the highest activity (28.3 ± 2.0 U/g), which was significantly superior to the control treatment (18.5 ± 1.3 U/g). The 5% EM treatment showed significantly higher activity than the CK treatment (difference = 9.8 U/g, *p* < 0.001) and was also significantly superior to the 10% EM treatment. The booting stage represented the peak phase of root activity. Activity gradually declined during flowering and maturity, although 5% EM treatment maintained relatively high levels (flowering: 24.1 ± 1.7 U/g; maturity: 19.5 ± 1.4 U/g). In contrast, the 15% EM treatment showed significantly reduced activity (flowering: 16.2 ± 1.1 U/g; maturity: 13.8 ± 0.9 U/g). The 5% EM treatment exhibited significantly higher activity than the 15% EM treatment, but showed no significant difference compared to the 10% EM group. The CK treatment showed no significant difference in activity compared to the 15% EM group, but the 5% EM group was significantly higher than the CK treatment; the 5% EM treatment maintained relatively high activity at maturity (19.5 U/g).

The yield per plant varied significantly among treatments (Figure 4F). Compared to the control (CK, 165.04 g), yield in the EM-treated groups exhibited a trend of initial increase followed by a decrease with increasing EM concentration. The 2% EM and 5% EM treatments yielded the highest values of 184.46 g and 207.49 g, respectively, representing increases of 11.8% and 25.7% over CK. Statistical analysis confirmed that the 5% EM treatment was significantly higher than both the CK and the 2% EM treatment (*p* < 0.05). Conversely, the 10% EM and 15% EM treatments showed declines to 201.83 g and 183.38 g, respectively, indicating that while appropriate EM application enhances grain yield, excessive concentrations diminish this effect. Figure 4G shows that the trend in 100-grain weight closely parallels that of yield-per-plant. The CK group recorded a 100-grain weight of 28.59 g. The 5% EM treatment yielded the highest value at 37.41 g, representing a 30.9% increase. The 10% EM treatment maintained a high level of 39.57 g, while the 15% EM treatment decreased to 33.48 g. This indicates that 5–10% EM treatment significantly promotes grain plumpness.

Aboveground dry matter weight was higher than the CK (366.55 g) in all treatments (Figure 4H), with the 0.5% EM treatment yielding the highest value at 429.60 g, representing a 17% increase. As EM concentration further increased, dry matter weight slightly decreased but remained significantly higher than that of the CK, indicating that EM treatment effectively promotes plant dry matter accumulation.

Regarding root weight (Figure 4I), the CK group averaged 87.49 g. All EM treatments exceeded CK, with the 5% EM treatment yielding the highest root weight at 108.20 g, representing a 23.7% increase. The 10% EM and 15% EM treatments yielded root weights of 106.39 g and 93.82 g, respectively, which remained higher than CK but lower than the 5% EM treatment, suggesting that moderate EM application aids root development.

The number of fibrous roots showed little variation among treatments (Figure 4J). The CK group had 44 roots, while EM treatments ranged from 43 to 53 roots, with the 5% EM treatment yielding the highest number (50.33 roots), indicating a relatively minor effect of EM treatment on lateral root number.

Stem diameter showed no significant differences among treatments (Figure 4K). The CK group measured 8.67 mm, while EM treatments ranged from 8.83 to 9.17 mm, indicating limited influence on stem diameter. Regarding plant height (Figure 4L), the CK group averaged 209.67 cm. The 0.5% EM treatment reached 214.33 cm, representing a 2.2% increase over CK; The 2% EM treatment measured 199.00 cm, a 5.1% decrease compared to CK; the 5% EM treatment measured 209.33 cm, essentially unchanged from CK (–0.2%); the 10% EM treatment measured 201.00 cm, a 4.1% decrease; and the 15% EM treatment measured 202.67 cm, a 3.3% decrease. Overall, except for the 0.5% EM treatment, the other treatments did not show a promoting effect on plant height, with some treatments even showing a decrease. This indicates that the impact on plant height through EM treatment was insignificant and, at high concentrations, even slightly inhibitory.

The 5% EM treatment consistently and significantly enhanced key yield components (total grain weight, 100-grain weight) and biomass (dry matter, root weight) relative to CK (Figure 4F–I). In contrast, its influence on plant morphological traits (lateral root number, stem diameter, plant height) was minimal, as these parameters showed little variation across treatments (Figure 4J–L). Efficacy declined at higher concentrations (15% EM), exhibiting even inhibitory trends, suggesting an optimal concentration range for EM application. Notably, EM treatment had limited effects on promoting above-ground plant height. Its yield-enhancing effects primarily stemmed from positive impacts on yield components (e.g., grain weight) and root development.

### 3.5. Correlations Between Substrate Environment, Lignocellulosic Content, Enzyme Activity, and Maize Yield

**Figure 5:**
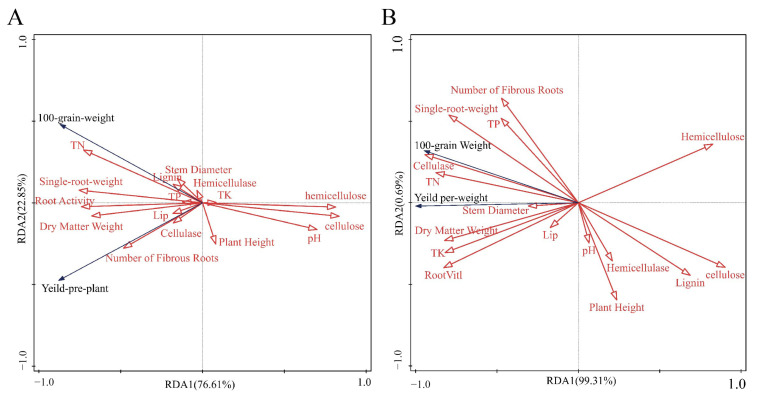
(**A**) Redundancy analysis of compost substrate properties and maize yield indicators; (**B**) Redundancy analysis of substrate properties and maize yield indicators across growth stages. Red arrows represent explanatory variables (pH, lignocellulose components, enzyme activities, nutrient contents, and plant growth parameters). Black arrows represent the response variables (yield-per-plant and 100-grain weight). The significance of explanatory variables was tested by Monte Carlo permutation (*p* < 0.05). The direction and length of arrows indicate the strength and sign of correlations with the ordination axes.

Figure 5A presents the results of Redundancy Analysis (RDA) based on environmental factors at the 30–day composting stage and maize yield traits (yield-per-plant and hundred-grain weight). Figure 5B displays the RDA based on environmental factors during the vegetative stage and yield indicators at maturity. Both studies indicate that environmental factors exert a significant influence on maize yield, revealing intrinsic relationships between environmental conditions at different stages and yield formation.

Figure 5A shows significant positive correlations between root vitality, dry matter accumulation, and total nitrogen (TN) content with maize yield indicators (yield-per-plant and 100-grain weight). These positive relationships were observed within the multivariate context shaped by the composite environmental factors. root vitality exhibited the highest variance explained, indicating its primary driving role in yield formation. This is facilitated by enhanced water and nutrient uptake through well-developed roots, which directly promotes grain filling. Conversely, significant negative correlations were observed between cellulose and hemicellulose content and yield, suggesting that higher degradation of fibrous materials in the compost substrate is more conducive to yield enhancement.

Analysis in Figure 5B further validates and deepens these trends. This figure not only reaffirms the positive contributions to yield from nutrient supply (e.g., total nitrogen, total phosphorus) and plant growth status (e.g., dry matter accumulation), but also reveals a strong positive correlation between cellulase activity and yield formation. This finding indicates that cellulase activity directly influences the degradation efficiency of organic materials (e.g., cellulose, hemicellulose) during composting. Efficient degradation is a crucial factor in enhancing the efficacy of compost products as fertilizers and improving soil nutrient availability. Therefore, cellulase activity can serve as a crucial microbiological indicator for evaluating compost quality and potential fertilizer efficacy.

Analysis of the two RDA models collectively identifies two major groups of key environmental factors influencing maize yield: nutrient availability (total nitrogen, total phosphorus, etc.) and the degree of organic matter degradation in the compost substrate (reflected by indicators such as cellulase activity and fiber content). This profoundly reveals the critical importance of optimizing the physicochemical properties and microbial activity of the compost substrate for enhancing maize yield.

The findings underscore the importance of managing the substrate environment (e.g., nutrient and water supply) during key growth stages (tasseling to flowering) to support the plant physiological processes essential for high yield, namely, high root vitality and substantial dry matter accumulation.

### 3.6. Correlation Between Compost and Maize Growth Indicators

**Figure 6:**
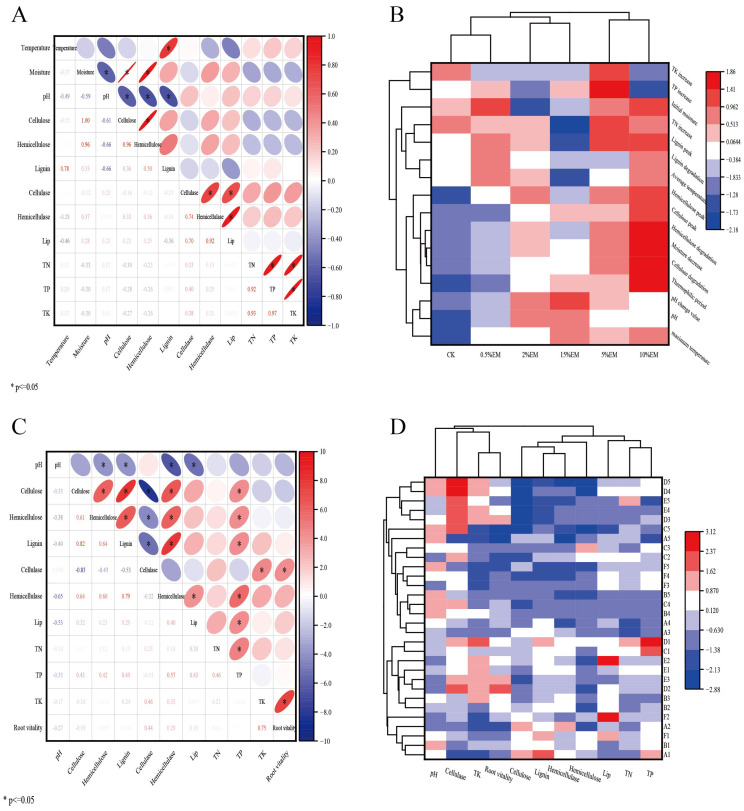
(**A**,**B**) Heatmap showing correlations between soil environmental factors, lignocellulose content, and enzymes during composting periods, the color scale represents the correlation coefficient (r), with red indicating positive correlations and blue indicating negative correlations. Asterisks denote significance levels (* *p* < 0.05); (**C**,**D**) Grouped cluster heatmaps showing the correlation patterns among substrate environmental variables and the plant physiological indicator (root vitality) during maize growth, the color scale represents standardized values (z-scores), where red indicates values above the overall mean and blue indicates values below the overall mean for a given variable across all samples; In Figure 6D, A1–A5 represent the seedling stage, jointing stage, tasseling stage, flowering stage, and maturity stage for the CK group; B1–B5 represent the seedling stage, jointing stage, tasseling stage, flowering stage, and maturity stage for the 0.5% EM group; C1–C5 represent the seedling stage, jointing stage, tasseling stage, flowering stage, and maturity stage for the 2% EM group; D1–D5 represent the seedling stage, jointing stage, tasseling stage, flowering stage, and maturity stage for the 5% EM treatment group; E1–E5 represent the seedling stage, jointing stage, tasseling stage, flowering stage, and maturity stage for the 10% EM group; F1–F5 represent the seedling stage, jointing stage, tasseling stage, flowering stage, and maturity stage for the 15% EM group.

Spearman correlation analysis was performed on composting fermentation period indicators (including temperature, moisture content, pH, cellulose, hemicellulose, lignin, cellulase, hemicellulase, lignin peroxidase, total nitrogen, total phosphorus, total potassium). The significance levels of correlation coefficients are marked as * *p* < 0.05 and ** *p* < 0.01. with results shown in Figure 6A. During fermentation, pH correlated negatively with moisture content (r = −0.667, *p* < 0.01), cellulose (r = −0.707, *p* < 0.01), hemicellulose (r = −0.615, *p* < 0.01), hemicellulase activity (r = −0.486, *p* < 0.01), and Lip activity (r = −0.498, *p* < 0.01). This suggests that pH is a crucial factor influencing the degradation of organic matter and enzyme activity during composting. Moisture content showed significant positive correlations with cellulose (r = 0.954, *p* < 0.01), hemicellulose (r = 0.819, *p* < 0.01), and lignin (r = 0.297, *p* < 0.05). Temperature showed significant positive correlations with lignin (r = 0.741, *p* < 0.01) and cellulase activity (r = 0.757, *p* < 0.01), indicating that elevated temperatures facilitate lignin degradation and enhance cellulase activity.

Figure 6B visualized the effects of different EM concentrations on the *Auricularia heimuer* residue-chicken manure composting process through hierarchical cluster analysis and a heatmap. As shown, the heatmap clearly reveals distinct response patterns across the treatment groups (CK, 0.5% EM, 2% EM, 5% EM, 10% EM, 15% EM) for 12 physicochemical and biological characteristics. The control group (CK) exhibits extensive blue areas, indicating that without EM inoculation, key indicators such as compost temperature characteristics, organic matter degradation rate, and enzyme activity were significantly below average, resulting in the lowest composting efficiency. In the hierarchical clustering analysis (Figure 6B), the CK, 0.5% EM, and 2% EM treatment groups were positioned within the same major cluster branch, suggesting that these low-concentration inoculation treatments elicited a more similar overall response in the measured composting parameters compared to the distinct patterns shown by the medium- and high-concentration EM treatments (5%, 10%, and 15%), demonstrating the most similar and efficient composting effects. Notably, the 10% EM group exhibited extreme red outliers in terms of thermophilic phase duration, cellulose and hemicellulose degradation rates, and moisture reduction rates, indicating the most intense and rapid composting reaction. The clustering pattern in Figure 6B indicated that the 15% EM group did not closely cluster with the 5% and 10% EM groups, despite some similarities in specific metrics. Its unique profile, particularly the extreme reduction in total nitrogen increase, sets it apart. This outlier separated it from the other groups in the clustering analysis. This suggests that excessive EM microbial concentration may have triggered severe nitrogen loss, producing an inhibitory effect.

Hierarchical cluster analysis of the composting parameters (Figure 6B) revealed distinct groupings among the indicators. Two prominent functional clusters were identified based on their statistical similarity and biological interpretation: (1) a ‘Degradation Efficiency Cluster’, comprising closely related dynamic process indicators (e.g., cellulose/hemicellulose degradation rates, thermophilic phase duration, moisture loss, peak cellulase activity); and (2) a ‘Nutrient Retention Cluster’, consisting of the final total phosphorus and potassium contents. The indicators within the ‘Degradation Efficiency Cluster’ exhibited high values in the 10% EM treatment, indicating strong positive correlations among them and collectively reflecting intense composting activity. In contrast, indicators in the ‘Nutrient Retention Cluster’ showed high values in the 5% EM treatment but lower values in the 10% EM treatment, suggesting a potential trade-off between rapid degradation and nutrient conservation. Additionally, the ‘Total Nitrogen Increase’ exhibited a unique and divergent response pattern, effectively separating itself from the other variables in the clustering structure and thus being treated as an independent, anomalous factor in the analysis, primarily due to its extremely negative values in the 15% EM treatment.

Figure 6C,D present Pearson correlation analysis and cluster heatmap analysis of maize growth stages, respectively, reflecting correlations among multiple physiological and biochemical indicators (e.g., pH, cellulose, hemicellulose, lignin, enzyme activity, nutrient elements, and root vitality) during different growth phases (seedling stage, jointing stage, tasseling stage, flowering stage, and maturity stage).

Organic matter degradation is closely linked to enzyme activity, exhibiting significant positive correlations among cellulose, hemicellulose, and lignin (r ≥ 0.598, *p* < 0.01). This indicates consistent trends in their degradation patterns as the primary organic components within the substrate. All three components showed a significant negative correlation with cellulase activity (r ≤ −0.489, *p* < 0.01), indicating that more thorough organic matter degradation occurs with higher cellulase activity.

Among the plant physiological traits analyzed, root vitality exhibited a highly significant positive correlation with potassium content (r = 0.744, *p* < 0.01), indicating that potassium plays a crucial role in promoting root development. This strong relationship highlights root vitality as a key integrative physiological response linking substrate potassium availability to overall plant performance. Root vitality also showed significant positive correlations with cellulase and hemicellulase activity (r = 0.394, 0.377, *p* < 0.05), further confirming the positive influence of enzyme activity on root health.

pH exhibited negative correlations with enzyme activities, showing significant negative correlations between pH and hemicellulase (r = −0.647, *p* < 0.01) and ligninase (r = −0.587, *p* < 0.01), indicating that these enzymes are more favorably expressed and active under acidic conditions.

Synergistic effects were observed among nutrient elements, with TP showing significant positive correlations with TN, hemicellulose, cellulose, and lignin. This suggests a close relationship between phosphorus availability in the substrate and other nutrients, as well as the degradation of organic matter.

Cluster analysis results showed that the CK group clustered with the low-concentration EM groups (0.5% EM, 2% EM), exhibiting an overall blue color (below average), indicating limited effects from low-concentration EM treatment. The medium to high EM concentration groups (5%, 10%) clustered together, exhibiting an overall red color (above average), and showed the best performance, especially from the booting stage to maturity. Although the 15% EM group shared some similarities with the 5% EM and 10% EM groups, it exhibited suppression in multiple indicators during the maturity stage (e.g., TK, root activity), suggesting that excessively high concentrations may have negative effects.

Indicator cluster analysis revealed: “Organic Matter-Enzyme Activity” cluster: Clustered together for cellulose, hemicellulose, lignin, cellulase, and hemicellulase, showing consistent response patterns. The “nutrients-root system” cluster grouped TN, TP, TK, and root vitality, indicating a close relationship between nutrient supply and root development. pH formed an independent cluster, exhibiting relatively independent variation patterns that were primarily influenced by EM concentration and growth stage.

During the composting phase, 5–10% EM treatment effectively elevates pile temperature, prolongs the thermophilic phase, promotes cellulose and hemicellulose degradation along with enzyme activity, and accelerates the composting maturation process. In contrast, the highest concentration tested (15% EM) led to inhibited nitrogen retention and a net loss of total nitrogen. During maize growth, substrate properties are closely linked to root development, with enzyme activity (cellulase and hemicellulase) exhibiting a significant negative correlation with organic matter degradation. The 5–10% EM concentration performs optimally from tasseling to maturity, significantly enhancing substrate nutrients (TN, TP, TK) and root vitality. Root systems and grain traits play a dominant role in yield formation. EM microbial concentration showed no significant direct correlation with yield indicators, indicating that its effects are primarily mediated through improvements in compost quality and substrate environment. Thus, inoculation with 5–10% EM concentration enhances substrate nutrient status and enzyme activity by optimizing the composting process, thereby promoting maize root development and grain filling, ultimately influencing yield. Excessively high concentrations (15% EM) may cause nitrogen loss and inhibitory effects.

During the composting phase, an EM concentration range of 5–10% is optimal for effectively raising pile temperature, prolonging the thermophilic phase, promoting cellulose and hemicellulose degradation along with enzyme activity, and accelerating compost maturation. Concentrations exceeding 15% EM may inhibit nitrogen retention, resulting in total nitrogen loss. During maize growth, substrate properties are closely linked to root development. Enzyme activity (cellulase and hemicellulase) exhibits a significant negative correlation with organic matter degradation. The 5–10% EM concentration performs best from tasseling to maturity, significantly enhancing substrate nutrients (TN, TP, TK) and root vitality. Yield formation is primarily governed by root system and grain traits. EM microbial concentration shows no significant direct correlation with yield indicators, indicating that its effects are mainly mediated through improvements in compost quality and substrate conditions. Thus, inoculation with 5–10% EM concentration enhances substrate nutrient status and enzyme activity by optimizing the composting process, thereby promoting maize root development and grain filling, ultimately influencing yield. Excessively high concentrations (15%) may cause nitrogen loss and inhibitory effects.

## 4. Discussion

### 4.1. Effect of EM on the Composting Cycle

#### 4.1.1. Effect of EM Inoculation on Temperature and Moisture Content

EM inoculation significantly shortened the initial heating phase of composting and prolonged the duration of the thermophilic phase, indicating its effective optimization of microbial community activity, promoting rapid temperature rise and stable maintenance within the compost pile. The 5–10% EM treatment groups exhibited more stable temperatures during the thermophilic phase and more thorough composting processes. This aligns with Guo et al.’s findings, which demonstrated that microbial inoculation increased the thermophilic phase temperature by 3–7 °C in the co-composting of SMS and chicken manure [25] and elevated the maximum temperature to over 65 °C in straw composting through the addition of exogenous microbes [26]. In this study, the thermophilic phase at ≥50 °C lasted 9–16 days across 0.5–15% EM treatments, aiding pathogen and weed seed inactivation to enhance compost sanitation [27]. The high-temperature thermophilic phase (52–60 °C) represents the optimal activity range for composting microorganisms [28]. By promoting thermophilic bacterial proliferation, EM not only elevated pile temperatures but also extended the high-temperature phase by 2–3 days, further accelerating organic matter decomposition.

The optimal moisture content (MC) for composting systems is 50–60% [29]. In this study, all treatment groups maintained an initial MC of 60%. During composting, the moisture content decreased more rapidly in the EM-treated groups, indicating more active microbial metabolism and higher rates of organic matter decomposition. This aligns with the effects of elevated temperature and ventilation accelerating moisture evaporation [30,31].

#### 4.1.2. pH Changes and Fermentation Process

During composting, pH undergoes a three-stage change characterized by “initial decrease, subsequent increase, and final stabilization,” consistent with the typical accumulation of organic acids and subsequent alkalization caused by ammonia volatilization in composting systems [32]. The addition of EM inoculants significantly influenced pH dynamics: 15% EM induced initial severe acidification (pH decrease of 19.7%), potentially suppressing some microbial activity [33]; 5–10% EM treatments rapidly rebounded to alkaline ranges during the middle phase and ultimately stabilized around pH 8.0, meeting mature compost standards (pH < 9) [34]. Composting microorganisms typically thrive within a pH range of 6.7–9.0 [35]. SMS compost supplemented with urea and chicken manure reached pH values of 8.5–9 by day 30 [36]. All treatments in this study ultimately achieved final pH values within this range, indicating that EM inoculation did not disrupt the microbial environment but rather optimized the fermentation process.

Throughout the maize growth period, soil pH exhibited an overall downward trend. The 5% EM treatment consistently showed the lowest pH values at all stages (6.90 at maturity), indicating a strong soil buffering capacity. This likely mitigated soil alkalization by promoting the metabolism of organic acids and microbial activity [37].

### 4.2. Promotion of Lignocellulose Degradation by EM Inoculation

#### 4.2.1. Degradation of Cellulose and Hemicellulose

The results of this study indicate that the addition of EM significantly promotes the degradation of cellulose and hemicellulose during composting, with the most pronounced effect observed at 5–10% EM concentration. This aligns with previous research findings that inoculation with exogenous microorganisms can effectively enhance the degradation efficiency of lignocellulose [38,39]. For instance, in maize stover-pig manure composting, microbial inoculation achieved degradation rates of 58.9%, 85.39%, and 51.58% for cellulose, hemicellulose, and lignin, respectively [40]; in peach wood chip composting, the cellulose degradation rate reached as high as 47.13% [41]. During the initial composting phase (1–3 days), cellulose degradation rates remained low across all treatment groups, likely due to microorganisms prioritizing soluble organic carbon (DOC) in the substrate. As DOC was consumed, pile temperature and microbial activity rapidly increased, triggering explosive growth in cellulose degradation rates between days 3–7 [42,43]. The 5–10% EM treatments maintained higher degradation rates during the high-temperature phase, further confirming their enhancing effect. Throughout the maize growth period, the 5% EM treatment exhibited the lowest cellulose content during tasseling, with overall degradation rates increased by 45.2–76.1%, demonstrating its highly efficient degradation capacity across different growth stages [44]. Although hemicellulose degradation fluctuated significantly, the 5% EM treatment still achieved the lowest value during the flowering stage, reducing it by 52.0% compared to the control, further illustrating the advantages of 5–10% EM in treating structural carbohydrates [45].

#### 4.2.2. Lignin Degradation

Lignin degradation primarily occurs during the later stages of composting, with 15% EM exhibiting a more substantial degradation-promoting effect. This aligns with Joergensen’s findings [46], indicating that 15% EM inoculants are more suitable for treating recalcitrant organic matter. Its absolute content was significantly lower than CK (*p* < 0.05), demonstrating that high-concentration EM inoculants positively influence the decomposition of recalcitrant components. Research indicates that lignocellulose degradation is closely linked to the formation of humic acid (HA). Aromatic compounds (e.g., phenols and quinones) released during lignocellulose breakdown undergo polymerization reactions with amino acids, reducing sugars, and polysaccharides, ultimately forming HA [47,48]. The accelerated lignin degradation observed in the 5% EM group during the flowering period may be attributed to the promotion of microbial enzyme activity by crop phenolic secretions. Research indicates that LiP plays a pivotal role in lignocellulose decomposition during biomass degradation and functionalization [49]. In this study, lignin peroxidase (LiP) activity significantly increased during the mid-to-late stages and showed a positive correlation with EM concentration, further substantiating its critical role in lignin degradation.

#### 4.2.3. Correlation Between Enzyme Activity and Degradation Efficiency

The degradation efficiency of lignocellulose is closely related to the activities of key enzymes. This study revealed that cellulase and hemicellulase activities significantly increased during the mid-to-late stages of composting. Specifically, cellulase activity peaked at day 7 in the 10% EM treatment, reaching 26.1% higher than the control (CK). The 5% EM treatment exhibited the highest enzyme activity during the maize tasseling stage, showing a 112.0% increase, indicating a significant positive correlation with degradation efficiency. This finding aligns with the results reported by Wang et al. [40].

Photosynthetic bacteria, lactic acid bacteria, and actinomycetes in EM not only suppress pathogens but also secrete highly active cellulase and hemicellulase, converting lignocellulose into small-molecule organic compounds and promoting humification in composting [50,51]. Notably, 15% EM concentrations may suppress enzyme activity during early composting due to microbial competition, as evidenced by the abrupt decline in hemicellulase activity on day 7. Furthermore, degradation capacity decreased during the late growth stage of maize, underscoring the critical importance of concentration regulation for maintaining enzyme activity.

Lignin peroxidase (LiP) plays a crucial role in disrupting the encapsulating structure of lignin around cellulose and hemicellulose [31]. In this study, 10–15% EM treatments significantly enhanced LiP activity during the jointing stage, while 5% EM maintained high levels during flowering and maturity. This suggests that EM components, such as white rot fungi and Bacillus species, may promote lignin depolymerization by secreting LiP [50], thereby providing viable microbial resources for the biological pretreatment of agricultural waste [52]. EM significantly accelerated lignocellulose degradation in compost and during maize growth by enhancing microbial enzyme activity, with 5–10% EM being the optimal concentration range. Both excessively high and low concentrations may reduce efficacy. Future studies should further elucidate the enzyme systems of key functional microorganisms within EM and their interaction mechanisms with indigenous microbial communities.

### 4.3. Effects of EM on Nutrient Retention and Transformation in Compost

The addition of EM significantly increased total nitrogen (TN), total phosphorus (TP), and total potassium (TK) content in the *Auricularia heimuer* Residue-chicken manure co-composting system, consistent with findings by Van Fan et al. [53]. The 5–10% EM treatment group demonstrated particularly pronounced effects, likely due to enhanced microbial nitrogen fixation within this concentration range, thereby reducing nitrogen loss. The use of microbial preparations increases microbial abundance and activity in soil [54], promoting organic matter decomposition and elevating TN content. EM also facilitates phosphorus solubilization and potassium release, though excessively high concentrations (e.g., 15% EM) may cause nutrient levels to decline during later composting stages. Notably, the 5–10% EM treatment maintained high nutrient levels throughout the entire composting process.

During composting, various forms of phosphorus, including total phosphorus (TP), available phosphorus (AP), water-soluble phosphorus (WSP), organic phosphorus, and inorganic phosphorus, undergo mutual transformations. However, total phosphorus content does not decrease due to volatilization [55]. Consistent with this finding, all treatment groups in this study exhibited increased total phosphorus content. This may be attributed to fertility activators within EM preparations, which potentially enhance phosphorus availability. Potassium release depends on silicate-solubilizing bacteria activity. The 5% EM treatment likely enhanced the potassium-solubilizing capacity of these bacteria, whereas the 15% EM treatment may have inhibited potassium release by disrupting bacterial structures due to high osmotic pressure [55,56].

Nitrogen, phosphorus, and potassium are critical nutrients for maize growth and development, with particularly high demands during root development, flowering, and grain formation stages. Numerous studies indicate that manure application significantly increases soil available nitrogen (AN), available phosphorus (AP), and available potassium (AK) levels, providing ample nutrients for maize growth [57,58]. In this study, inoculated treatments exhibited higher TN, TP, and TK levels in the substrate compared to the control group, better meeting plant nutrient uptake requirements.

### 4.4. Effects of EM on Maize Yield

Regarding maize growth and yield, the increase in yield was primarily attributed to higher grain weight and improved grain filling. The 5% EM treatment led to a significant increase in yield per plant compared to the control. 100-grain weight was substantially increased by the 2%, 5%, and 10% EM treatments relative to the control, with no significant differences observed among these three EM levels. Similarly, studies indicate that incorporating straw-insect manure compost into soil significantly enhances maize plant height, biomass, root vitality, total leaf phosphorus content, and net photosynthetic rate. However, excessive application (e.g., 6% straw) may inhibit growth, potentially due to elevated salinity [59].

EM compost treatment also promotes growth parameters in various crops (e.g., bok choy [60], chili peppers [61], sesbania [62], bean [63], strawberry [64]). In legumes, EM treatment significantly increased stem height, stem weight per plant, leaf area, number of leaves, root length, root dry weight, number of seeds per plant, and seed weight [13,20]. Furthermore, the interaction between EM and biochar significantly promoted germination, plant height, stem diameter, root biomass, aboveground biomass, and total biomass in hemp seeds [16]. Combined EM inoculants substantially enhance soil microbial activity, thereby improving soil nutrient utilization [65]. Studies indicate that EM composite inoculants increased chili pepper yield by 173.40% [66].

Composting rice straw and poultry manure inoculated with EM microbial communities significantly increases soil organic carbon, humus, and available nitrogen content, thereby comprehensively enhancing soil fertility. Applying EM compost to flower cultivation enhances soil microbial activity and enzyme system function, promoting nutrient cycling and plant growth [11]. This demonstrates not only its potential for enhancing nutritional value but also its practical advantages in agricultural production.

Correlation analysis in this study further revealed a positive relationship between maize yield at maturity and root vitality, consistent with findings by Lei et al. [67]. Considering composting efficiency, nutrient release, enzyme activity, and maize growth indicators, the 5% EM concentration emerged as the optimal treatment. It demonstrated the best performance in optimizing the composting process (evidenced by reduced heating time and extended thermophilic duration), promoting lignocellulose degradation, increasing nutrient content, and boosting maize yield. By introducing diverse functional microorganisms, EM inoculants enhanced key enzyme activities (cellulase, hemicellulase, and lignin peroxidase) as directly measured in this study. This accelerated the decomposition of organic matter (particularly lignocellulose) and the release of nutrients (TN, TP, TK), thereby improving compost quality and soil fertility, and ultimately promoting maize growth and yield formation.

The application of EM in the *Auricularia heimuer* Residue-chicken manure composting system not only enhances the resource utilization efficiency of agricultural waste but also significantly improves the quality of compost products and subsequent crop yields, demonstrating broad application prospects in green agriculture and sustainable development. This study preliminarily identified the optimal concentration and action mechanism of EM in *Auricularia heimuer* Residue-chicken manure co-composting and maize growth. However, limitations remain, such as the lack of investigation into deeper mechanisms like microbial community structure changes and functional gene expression. While this study confirmed our initial hypotheses, the underlying molecular mechanisms remain unclear. Future multi-omics research is needed to elucidate how EM regulates composting and crop growth.

Returning to the hypotheses proposed at the outset of this study, our findings provide substantial evidence supporting each of them. First, EM inoculation did significantly accelerate the composting process (reduced heating time, prolonged thermophilic phase) and improved compost quality (enhanced lignocellulose degradation and nutrient retention), confirming hypothesis (1). Second, the 5–10% EM concentration range was indeed optimal for promoting lignocellulose degradation and nutrient release, with the 5% treatment offering the most balanced benefits, which aligns with hypothesis (2). Finally, the application of EM-treated compost significantly increased key soil enzyme activities (cellulase, hemicellulase), which in turn promoted maize root development, growth, and yield formation, thereby validating hypothesis (3). The consistent positive correlations between enzyme activity, root vitality, nutrient availability, and final yield, as revealed by RDA and correlation analyses, further solidify the mechanistic pathway implied in the initial hypotheses

## 5. Conclusions

This study systematically evaluated the effects of different EM concentrations on the co-composting process of *Auricularia heimuer* residue and chicken manure, as well as on maize growth. The following conclusions were drawn: The addition of EM significantly accelerated the composting process, increased pile temperature, prolonged the thermophilic phase, accelerated lignocellulose degradation, and optimized compost quality. The optimal EM addition concentration range was determined to be 5–10%. The 5% EM treatment demonstrated the best overall performance in composting efficiency, nutrient retention, and maize yield enhancement. It significantly increased soil total nitrogen, phosphorus, and potassium contents, enhanced key enzyme activities, and promoted maize root development and grain filling. Application of EM-treated compost significantly improved maize root vitality, yield-per-plant, 100-grain weight, and dry matter yield. The yield increase was primarily attributed to enhanced grain filling and root development. Redundancy and correlation analyses further revealed significant positive correlations between root vitality, total nitrogen content, cellulase activity, and yield. In summary, EM effectively improved the rhizosphere microenvironment and enhanced maize yield and quality by regulating compost microbial activity, boosting enzyme activity, and promoting nutrient conversion. This study provides a feasible technical pathway and theoretical support for the resource utilization of agricultural waste and green high-yield maize cultivation.

## Data Availability

The original contributions presented in this study are included in the article. Further inquiries can be directed to the corresponding authors.
